# Mothers’ experiences of unwanted early cessation of breastfeeding: a scoping review

**DOI:** 10.1186/s13006-026-00883-0

**Published:** 2026-08-12

**Authors:** Julie Mahon, Camilla Ejlertsen, Pia Dreyer, Sara R. Francis

**Affiliations:** 1 Department of Public Health, Aarhus University, Aarhus, Denmark; 2 Department of Anaesthesiology and Intensive Care, Aarhus University Hospital, Aarhus, Denmark; 3 Department of Gynaecology and Obstetrics, Aarhus University Hospital, Aarhus, Denmark

**Keywords:** Breastfeeding cessation, Breastfeeding difficulties, Early weaning, Emotional impact, Maternal experiences, Maternal identity, Postnatal care, Scoping review

## Abstract

**Background:**

Many mothers intend to breastfeed but stop earlier than planned, despite strong public health recommendations from the World Health Organisation promoting exclusive breastfeeding for the first six months of life. Although early cessation is widespread, it remains poorly understood, particularly regarding how mothers experience stopping earlier than intended and how these experiences shape their sense of self, identity, and motherhood. Existing research has primarily focused on prevalence and predictors, with less attention to mothers’ lived experiences. This scoping review aimed to map existing knowledge of mothers’ experiences of unwanted early cessation of breastfeeding.

**Main body:**

The review followed the Joanna Briggs Institute methodology for scoping reviews. A comprehensive search was conducted in CINAHL, PubMed, PsycINFO, and Embase in June 2024, updated in July 2025 and updated again in June 2026. Reference lists and citations of relevant studies were also screened to ensure comprehensive coverage. Fifteen studies met the inclusion criteria. The findings were synthesised into three overarching themes: (1) The Ideal of the ‘Good Mother’ Under Pressure, (2) Existential Consequences Arising from Breastfeeding Difficulties, and (3) Insufficient Support Undermining Breastfeeding. Across the included studies, mothers described a pronounced gap between expectations and lived experiences, often accompanied by guilt, shame, and a pervasive sense of failure. Early cessation was not merely a practical outcome but a deeply emotional, identity-shaping event. These experiences were frequently intensified by perceived inadequacies in professional support, including a lack of individualised, consistent, and empathetic care, as well as conflicting advice from healthcare professionals.

**Conclusion:**

Unwanted early cessation of breastfeeding is a complex, emotionally significant experience that extends beyond feeding practices. This review offers a comprehensive overview of how mothers experience early cessation and highlights the need for breastfeeding support that is not only clinically and technically competent but also emotionally attuned and responsive to mothers’ needs. Recognising the existential and identity-related dimensions of early cessation may contribute to more responsive postnatal care and better support for mothers navigating this transition.

**Supplementary Information:**

The online version contains supplementary material available at 10.1186/s13006-026-00883-0.

## Introduction

The World Health Organisation (WHO) and the United Nations Children’s Fund (UNICEF) recommend exclusive breastfeeding during the first six months of life, without the introduction of any additional food or liquid, followed by the introduction of nutritionally adequate and safe complementary foods at six months alongside continued breastfeeding up to two years of age or beyond [40, 43]. A substantial body of evidence supports that breastfeeding is optimal for infant health, development, and nutrition, providing lifelong benefits for both infants and mothers [5, 11, 17, 21, 41]. Although most mothers initiate breastfeeding shortly after giving birth, many discontinue within the first six months, and only about 48% of infants aged 0–6 months are exclusively breastfed worldwide [44, 45].

A considerable body of literature explores the challenges mothers encounter when establishing and maintaining breastfeeding. Early difficulties, such as severe breast or nipple pain, problems with achieving a proper latch, concerns about milk supply, and insufficient practical or emotional support, often cause mothers to discontinue breastfeeding earlier than intended [4, 8, 10, 26, 28].

During pregnancy, many mothers plan to breastfeed exclusively for the recommended six months. When expectations are not met, mothers often experience significant disappointment and must adapt to alternative feeding methods [20]. Despite the high prevalence of unwanted early cessation of breastfeeding, defined here as cessation within the first six months postpartum that occurred earlier than the mother had wished or intended, limited literature addresses mothers’ lived experiences of this phenomenon. These experiences often go unrecognised in postnatal care and support systems, despite their prevalence. Existing studies indicate that unwanted early cessation of breastfeeding is frequently associated with emotional distress, guilt, and shame related to not meeting global public health recommendations for breastfeeding duration or mothers’ own aspirations and intentions [14, 20, 42, 46]. Furthermore, research shows that mothers who are unable to breastfeed may experience an ideal-actual discrepancy, as their expectations of breastfeeding come into conflict with the lived reality of not being able to breastfeed, which may predict postnatal guilt, shame, depression and anxiety [7, 22, 35, 37]. One study also found that greater reliance on formula feeding was linked to increased maternal guilt and higher postnatal anxiety levels [15]. Another study reported that early cessation of breastfeeding and postpartum depressive symptoms were significantly associated; multivariate logistic regression analysis showed that depressive symptoms were independently associated with early breastfeeding cessation [30]. These findings highlight the need for further inquiry into mothers’ experiences of unwanted early cessation of breastfeeding and the ways in which such experiences may shape maternal identity, emotional well-being, and future breastfeeding trajectories. A preliminary search of databases, including PubMed, CINAHL (EMBASE), PsycINFO (EBSCO), Embase (via Elsevier), and the Joanna Briggs Institute (JBI) Database, was performed to prevent evidence duplication [33]. No current or ongoing scoping reviews or systematic reviews specifically addressing mothers’ experiences of unwanted early cessation of breastfeeding were identified. While some reviews have focused on interventions to promote breastfeeding [38], none have examined the subjective experiences of mothers who stopped breastfeeding earlier than planned. This gap in the evidence base underscores the need for a systematic mapping of existing literature. Accordingly, this scoping review aims to synthesise the available literature on mothers’ experiences of unwanted early cessation of breastfeeding to provide a comprehensive overview and identify knowledge gaps for future research.

## Method

### Design

**Fig. 1:**
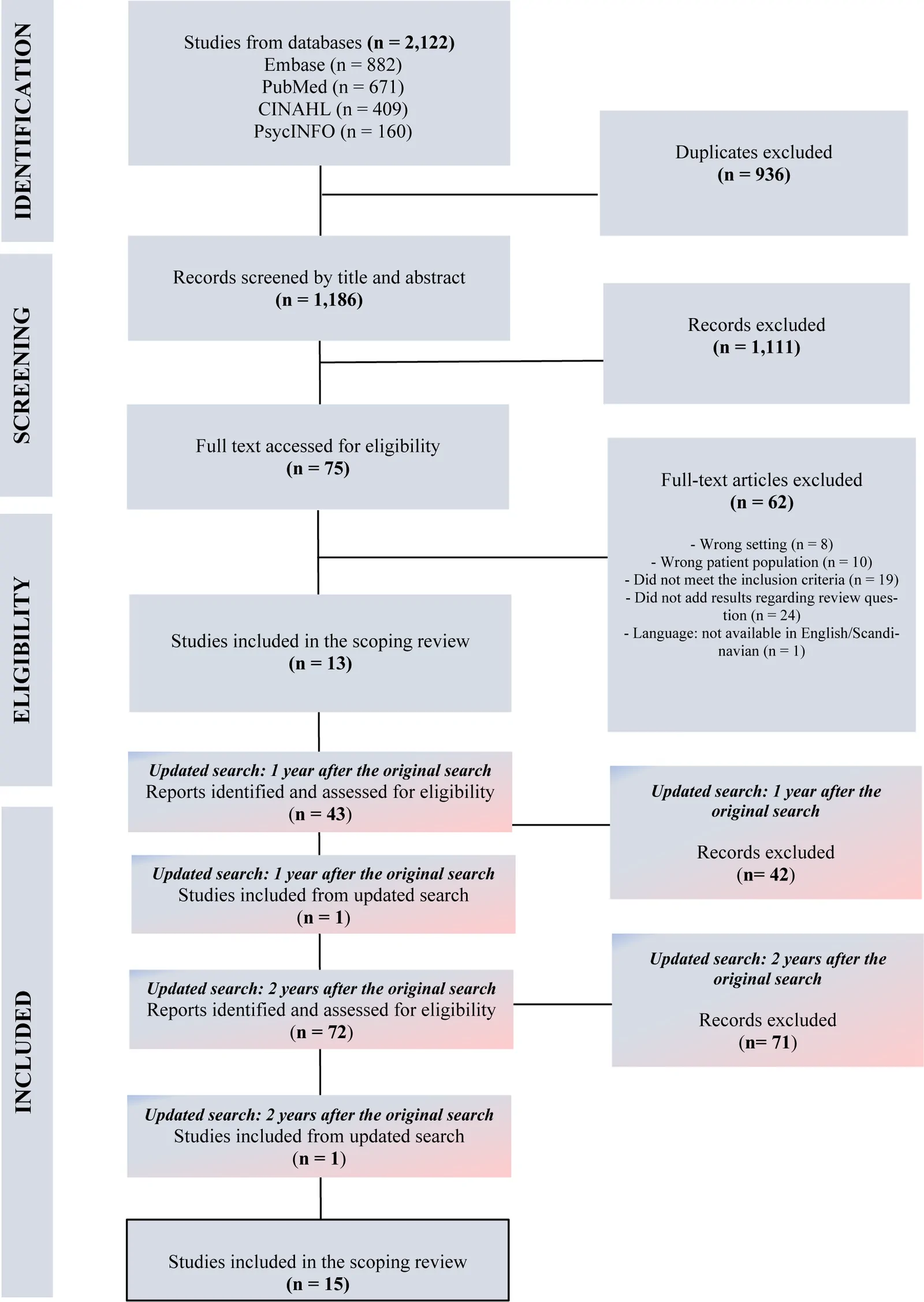
Preferred reporting items for systematic reviews and meta-analyses flow diagram of the systematic search strategy

The protocol for this scoping review was published previously [25]. The review was conducted in accordance with the Joanna Briggs Institute’s (JBI) methodology and guidance for scoping reviews and scoping review protocols. The process followed the recommended stages: identifying the research question; identifying relevant studies; selecting studies; charting data; and collating, summarising, and reporting the results [33]. Articles using diverse methodologies and perspectives were included - qualitative, quantitative, and mixed-methods designs - as well as studies from different disciplinary contexts exploring mothers’ experiences of unwanted early cessation of breastfeeding. The review aim and research question were structured according to the Population, Concept, and Context (PCC) framework (Appendix II) [33, 39]. The Preferred Reporting Items for Systematic Reviews and Meta-Analyses extension for Scoping Reviews (PRISMA-ScR) [39] was also applied to ensure transparent and rigorous reporting (see Fig. 1).

### Search strategy

The JBI’s recommended three-step search strategy was followed [33]. First, a limited, initial search of PubMed and CINAHL (EBSCO) was undertaken by the first author to identify text words and index terms in titles and abstracts. The findings from this preliminary search were used to develop the final comprehensive search strategy. Next, a comprehensive search was conducted in June 2024 using the databases CINAHL (EBSCO), PubMed, PsycINFO (via EBSCO), and Embase (via Elsevier), followed by an updated search in July 2025. A further literature search update was conducted in June 2026 using the same search strategy and language filters as in the previous searches. All identified keywords and index terms were applied across all included databases and adapted to the specific indexing systems of each.

Finally, forward and backwards citation tracking was performed, and the reference lists of included studies were screened for additional relevant articles [33]. The final search strategy was reviewed and validated by a research librarian who optimised the search design and ensured completeness. Two updated searches, one and two years later, ensured inclusion of the most recent evidence.

### Inclusion and exclusion criteria

The scoping review considered peer-reviewed original studies within qualitative, quantitative, and mixed-methods study designs for inclusion, with no restrictions regarding geographical location or cultural contexts. No lower publication year limit was applied; studies were eligible if they met the inclusion criteria and were identified in the searches conducted up to the final updated search in June 2026. Studies were eligible if they involved mothers ≥ 18 years (with minor exceptions described in Sect.  3, Findings) who had given birth at full term (≥ 37 weeks’ gestation) and whose infants were healthy (Population). Eligible studies were those that explored, described, referred to, or mentioned such mothers’ experiences of unwanted early cessation of breastfeeding within the first six months postpartum (Context). For this review, “early” was defined as cessation of breastfeeding within the first six months postpartum, while “unwanted” referred to cessation that occurred earlier than the mother had wished or intended. Studies were eligible for inclusion if they explored mothers’ experiences of breastfeeding cessation within this timeframe and described cessation as unwanted, unintended, or earlier than expected from the mother’s perspective.

Articles published in English and Scandinavian languages were included. Literature reviews and studies that did not examine mothers’ own experiences of unwanted early cessation of breastfeeding were excluded.

**Table 1:** Extraction of eligible studies (*n* = 14)

Authors (year) & Origin	Aim	Number of participants	Context	Methods	Main findings	Author’s interpretations
Mozingo, Droppleman and Merideth, [29] USA	To investigate the lived experiences of women who initiate breastfeeding but stop within the first two weeks after birth.	Nine mothers	Mothers who stopped breastfeeding within two weeks after initiating breastfeeding with a full-term infant.	Qualitative study. Interviews.	The women felt guilty for ceasing breastfeeding, as they considered “the good mother” ought to breastfeed. Some also felt guilty for feeling relieved that the struggle was over.	The women described a clash between highly idealised expectations and early breastfeeding problems. This led to incremental disillusionment and cessation of attempts. They spoke poignantly about failure, guilt, or shame, along with lingering self-doubt about not continuing.
Bailey et al. [3] UK	To examine cultural expectations and experiences of breastfeeding among first-time mothers from low-income areas.	16 first-time mothers	Mothers (from low-income areas) who had intended to breastfeed.	Qualitative study. Interviews.	The women were situated within a ‘give-it-a-go’ culture, expressing prenatal desire to breastfeed but perceiving failure as a realistic possibility.	Mothers who struggled with breastfeeding and later introduced formula milk expressed disappointment and guilt. Many sought recognition for their efforts and justification for their difficulties.
Redshaw and Henderson, [34] UK	To understand what is needed in the early days to enable breastfeeding initiation and continuation.	534 mothers	Mothers who gave birth in a single week in March 2006.	Mixed method. A survey with quantitative questionnaires and open-text responses.	Mothers often experienced a mismatch between their expectations and actual experiences. Most had decided to try breastfeeding, but when facing difficulties and wishing to give a bottle, they felt pressured by healthcare professionals. Unsuccessful breastfeeding affected how they perceived themselves as mothers.	Many women who succeeded felt they had “learned the hard way”, and some of those who did not felt that they were perceived as “bad mothers” who had “failed” at one of the earliest tasks of motherhood.
Kronborg, Harder and Hall, [19] Denmark	To explore mothers’ early breastfeeding experiences.	108 mothers	Mothers who gave birth to full-term singletons and initiated breastfeeding.	Mixed method. Two questionnaires, mainly with quantitative components, one with an open-text opportunity.	A fragility appeared that made the mothers vulnerable to their surroundings. The women felt breastfeeding pressure and were left with the sense of failing as mothers if they gave up breastfeeding.	Breastfeeding was closely linked to mothering and getting to know the baby. Being able to breastfeed the child seemed important to most of these mothers; their perception of themselves as new mothers hinged on how the breastfeeding progressed.
Leurer and Misskey, [23] Canada	To understand why early cessation remains common despite high initiation rates.	191 mothers	Mothers who had initiated breastfeeding and had infants aged 6 to < 12 months.	Mixed method. Survey with quantitative and qualitative components.	Breastfeeding evoked strong emotions. Regret, sadness, and guilt were expressed when breastfeeding ended earlier than desired.	Mothers often ceased breastfeeding due to unresolved problems, later expressing deep regret.
Massov, [27] New Zealand	To explore the breastfeeding experiences and perspectives of clinically overweight and obese women, and to gain an understanding of what influenced their infant feeding decisions	Six overweight or obese mothers	Mothers who initiated but did not continue exclusive or full breastfeeding.	Qualitative study. Interviews.	Women believed in the health benefits of breastfeeding but faced physical and psychological challenges leading to early cessation.	The study emphasises the importance of tailored antenatal and postnatal support for overweight and obese women.
Palmér, Carlsson, Brunt and Nyström, [32] Sweden	To explain and understand how the decision to continue or cease breastfeeding is influenced by the meaning of severe initial breastfeeding difficulties.	Eight mothers	Mothers who had experienced severe difficulties with initial breastfeeding.	Qualitative study. Interviews.	Mothers felt overtaken and violated by their own infants, bodies, and external expectations, leading to an existential crisis when deciding whether to continue or stop.	The study highlights the complex cultural and biological context of breastfeeding and its significant existential implications.
Hunt and Thomson, [12] UK	To explore why breastfeeding women do not access peer support.	13 mothers Additionally, 20 other participants: Health Professionals (*n* = 6). Peer supporters (*n* = 14), see Sect. 4. Findings for more information*.*	Mothers who breastfed for 5 + days without accessing peer support.	Qualitative study. Interviews. Focus group.	Mothers experienced pressure and judgment within a dichotomous landscape of infant feeding language and support.	Perceptions of judgment prevented access to support. Women often felt guilty and a sense of failure when difficulties arose.
Keevash et al. [16] UK	To understand the experiences of breastfeeding women and what affects their ability to continue breastfeeding.	41 mothers	Mothers who had breastfed for any period within the preceding five years.	Qualitative study. Interviews.	Breastfeeding difficulties acted as a stressor, hindering infant attachment. Emotional distress emerged when expectations to breastfeed were unmet.	Mothers who could not, or struggled to, breastfeed experienced emotional distress if they had expected to succeed. Many felt that they had failed to meet the expected standards. For some, breastfeeding was associated with being a good mother.
Ayton, Tesch and Hansen, [2] Tasmania, Australia	To explore how women experience cessation of exclusive breastfeeding.	127 mothers	Mothers with children aged 0–36 months.	Multimethod study. Questionnaires. Focus group. Interviews.	Cessation of exclusive breastfeeding triggered grief and a sense of failure, making it potentially harmful to women’s emotional well-being.	Mothers viewed breastfeeding as natural and best, and formula as wrong and unnatural. To avoid formula and prolong exclusive breastfeeding, they endured multiple issues. Cessation was often unexpected and devastating, leaving them with breastfeeding grief - a prolonged sense of loss and failure.
Cortés-Rua and Díaz-Grávalos, [6] Spain	To explore the personal experience and emotions of primiparous women who had to abandon breastfeeding before the expected time.	15 first-time mothers	Mothers who ceased breastfeeding before 4 months.	Qualitative study. Interviews.	Breastfeeding was perceived as both satisfying and sacrificial, yet also painful. Mothers experienced guilt and sadness, feeling they were worse mothers for having stopped.	Insecurity, external influences, and myths contributed to the introduction of the formula and ultimately led to its early cessation. Emotional ambivalence was prevalent.
Jackson, Mantler, and O’Keefe-McCarthy, [13] Canada	To gain a better understanding of the phenomenon of breastfeeding-related pain, how women experience this pain, and the meaning it holds for them.	14 mothers	Mothers who experienced breastfeeding-related pain in the past two months (included within 12 months postpartum)	Qualitative study. Interviews.	Women used enabling and barrier-reduction strategies to manage pain. Pain influenced perceptions and outcomes of breastfeeding.	Before giving birth, the women were unaware of breastfeeding-related pain and neither expected nor prepared for it. Those who stopped due to pain associated this with guilt and inadequacy, despite attempts to continue breastfeeding.
Spannhake, Jansen, Görig, Diehl, [36] Germany	To explore how mothers who have difficulties in breastfeeding perceive their situation and how they actively manage it.	15 mothers	Mothers who had unwanted breastfeeding cessation or problems.	Qualitative study. Interviews.	Women felt mentally overwhelmed and emotionally distressed when ‘forced’ to stop breastfeeding.	Negative emotions such as guilt, shame, and self-doubt were common and perceived to impact the mother–infant bonding.
Mahon and Dreyer, [24] Denmark	To explore how primiparous women experienced an unwanted early cessation of breastfeeding.	Five mothers	Mothers who stopped breastfeeding within the first six months.	Qualitative study. Interviews.	The mothers expressed concerns about what others might think of them as non-breastfeeding mothers, feeling that they did not meet the expectations of motherhood. Furthermore, breastfeeding was seen as an emotionally ‘hard fight’ that left the mothers feeling defeated.	Cessation led to grief, inadequacy, and weakened maternal identity. Support was perceived as insufficient before and after stopping.
Al-Marzouqi, Momani and Al-Dhoani, [1] Oman	To investigate the experiences of primiparous Omani mothers who practised exclusive breastfeeding.	35 first-time mothers	Mothers who had stopped breastfeeding within the first six months.	Qualitative study. Interviews.	Mothers began with optimism, viewing breastfeeding as natural and manageable. However, difficulties with positioning, pain, perceived low milk supply, inconsistent professional support, and limited family assistance beyond 6 weeks contributed to early discontinuation.	First-time mothers’ expectations of breastfeeding as instinctive were challenged by unanticipated barriers and insufficient support.

### Data extraction and analysis

Three independent researchers extracted data from the included studies. The retrieved studies from the systematic search were imported into the Covidence systematic review software (Veritas Health Innovation, Melbourne, Australia). The data extraction process involved an initial independent screening of titles and abstracts of all identified studies (*n* = 1,186). Studies that could not be resolved individually were discussed collectively with reference to the review aim and inclusion criteria. This was followed by an independent full-text screening of potentially eligible studies (*n* = 75). Once again, any discrepancies were discussed until consensus was reached, resulting in the inclusion of 13 studies. In the updated search in July 2025, one additional study was included, and in the updated search in June 2026, another was included, resulting in 15 studies in total.

Descriptions and accounts of mothers’ experiences of unwanted early cessation of breastfeeding were charted, extracted, and subsequently categorised in accordance with JBI methodology [33]. The analysis identified three overarching themes, presented in Sect. 3 (Findings).

## Findings

A total of 2,122 records were identified through the systematic search. After removing duplicates.

(*n* = 936), 1,186 records remained for title and abstract screening. Following the application of the inclusion and exclusion criteria, 1,111 records were excluded. A total of 75 studies were then assessed for eligibility, and 62 were excluded for not meeting the inclusion criteria. Thirteen studies were therefore included, as shown in the PRISMA flow diagram (Fig. 1). One year after the original search, an updated systematic literature search was conducted before submission to ensure the inclusion of all relevant studies. The first follow-up search identified 43 additional records, of which 42 were excluded for not meeting the inclusion criteria. One additional study [24] met the inclusion criteria and was subsequently included in the review (see Fig. 1). The second follow-up search identified 72 additional records, of which 71 were excluded for not meeting the inclusion criteria. One study [1] met the inclusion criteria and was subsequently included in the review (see Fig. 1).

Three included studies deviated slightly from the predefined inclusion criteria, but these deviations were deemed methodologically insignificant. All three studies were considered to provide valuable perspectives on the phenomenon of interest and were therefore retained. Two studies recruited mothers aged ≥ 16 years [2, 34], while one study also involved 13 mothers and 20 other participants, including healthcare professionals (*n* = 6) and peer supporters (*n* = 14) [12].

The included studies were summarised, and the most relevant information is presented in Table 1. The studies originated from Denmark [19, 24], Sweden [32], Germany [36], The United Kingdom [3, 12, 16, 34], Spain [6], Oman [1], Canada [13, 23], USA [29], Australia [2], and New Zealand [27]. All included studies were published between 2000 and 2026. 11 studies employed a qualitative design [1, 3, 6, 12, 13, 16, 19, 24, 27, 29, 32, 36]

Three studies had a mixed methods design: two were based on quantitative questionnaires with open-text responses [19, 34], one combined quantitative and qualitative components within a single questionnaire [23], and one study used a multimethod design incorporating questionnaires, focus groups, and interviews [2].

The mapping of study contents revealed three overarching themes related to mothers’ experiences of unwanted early cessation of breastfeeding: *(1) ‘The Ideal of the ‘Good Mother’ Under Pressure*,* (2) Existential Consequences Arising from Breastfeeding Difficulties*,* and (3) Insufficient Support Undermining Breastfeeding’* (Table 2).

### The ideal of the ‘good mother’ under pressure

In the first overarching theme, it was evident across the studies that mothers experienced pressure to breastfeed, arising both from societal norms and from internalised expectations.

Ayton et al. [2], Bailey, [3], Cortés-Rúa, [6], Hunt, [12], Jackson, [13], Keevash, [16], Kronborg, [19], Massov, [27], Mozingo, [29], Palmér, [32], Spannhake, [36]. One mother articulated this dual pressure:

There is a lot of pressure just in general society to breastfeed, a lot of it (…) I think mothers put a lot of pressure on themselves and cause a lot of problems to themselves because they can’t do it or they feel bad because they supplement their babies… I think we put a lot of pressure on ourselves to do it. I think it’s peer pressure to perform as a good mother… I did feel pressured to be a good mother and that and, like, you should breastfeed. ([27], p. 27)

Across the included studies, breastfeeding was described as closely connected to ideals of being a ‘good mother’ [2, 16, 27, 29, 32]. Consequently, breastfeeding becomes a significant marker of maternal success and a key aspect of early mothering ([19], p. 84). Across several studies, mothers expected breastfeeding to be instinctive, natural, and easy; yet, many felt unprepared for the physical and emotional realities of the experience [1, 3, 6, 12, 13, 27, 29, 32].

When breastfeeding proved unsuccessful, mothers described a dissonance between their idealised expectations and lived experiences ([2], p. 6). This maternal dissonance led to further emotional distress, notably as the inability to breastfeed conflicted with socio-cultural norms underpinning the ideal of motherhood ([29], pp. 8–12).

Mothers also reported fearing social judgment because they felt that they did not conform to the expected image of “the good mother” [24, 32, 36]. One mother expressed this fear:

I was made to feel (…) like I was a bad mother… and felt that I had somehow failed… (…) I still feel like I haven’t given my children the best start in life and feel like I lack something as a mother. ([34], p. 26)

Moreover, when mothers were unable to breastfeed and had to resort to formula feeding, they often perceived that formula milk symbolically replaced their milk, their breast, and thereby their maternal role, leaving them feeling redundant and diminished as mothers [2].

### Existential consequences arising from breastfeeding difficulties

The second overarching theme addressed how becoming a mother and the early cessation of breastfeeding were accompanied by profound existential consequences. The mothers expressed feelings of insecurity and vulnerability when experiencing breastfeeding difficulties in early motherhood [2, 6, 19, 32].

When breastfeeding proved unsuccessful, mothers reported feeling lost and emotionally burdened and disconnected from their maternal identity [12, 19, 29, 32, 36]. Additionally, mothers who were unable to breastfeed their infants reported guilt and shame [2, 12, 23, 24, 29, 32]. One mother reflected:

I wanted to feel that closeness, and I wanted to feel mothering. And I felt guilty for so long ([29], p. 21)

Furthermore, mothers who were unable to breastfeed or who experienced difficulties doing so felt that they were failing to meet the moral and cultural standards expected of them as mothers [16]. One mother stated:

I felt it was all tied up in my worth as a mother… this is about whether or not you’re a good mum. ([16], p. 656)

Similarly, several studies described how mothers perceived themselves as ‘bad mothers’ because of their inability to breastfeed [2, 6].

Regarding the early cessation of breastfeeding, several studies highlighted feelings of self-doubt, sadness, and disappointment as key existential consequences [2]; [23, 29, 36]. Grief frequently accompanied unsuccessful breastfeeding, as one mother expressed:

I think there was a whole grieving process for me around that, around letting go of that dream of this lovely relationship that’s going to happen. ([2], p. 6)

Another mother emphasised how grief was often unacknowledged within standard postpartum care:

Unsuccessful breastfeeding is often overlooked and ignored by others, because nobody has died and there is a common understanding that you can easily give your child a bottle with formula, and the problem is solved… But it does not change the fact that I, as a mother, feel grief about not being able to breastfeed my child as I had hoped and wished ([24], p. 4)

Moreover, several studies reported that mothers experienced a persistent sense of failure, both as women and as mothers, linked to the early cessation of breastfeeding [2, 12, 19, 24, 29, 32].

### Insufficient support undermining breastfeeding

The final overarching theme concerned breastfeeding support from healthcare professionals. Across several studies, mothers found that the support provided by healthcare professionals often fell short, being insufficient and poorly tailored to their individual needs [2, 6, 12, 16]. Overall, mothers reported that they were not adequately provided with enough information about what to expect from breastfeeding, including common challenges and practical strategies for overcoming them [36]. They were also frustrated by the lack of coherence and continuity in breastfeeding guidance and expressed a desire for a more consistent and standardised approach ([1, 16], p. 653).

Some mothers described a dichotomy in how support was delivered, with a prescriptive ‘these are the rules’ approach that conveyed the impression that there was only one correct way to breastfeed. This message was often internalised by mothers [12] and contributed to self-blame when they deviated from guidance. As noted by the study authors:

There was no notion of what might be ‘right’ for one mother might not be ‘right’ for another or that ‘the answer’ might need to be personalised or adapted. This led women to place themselves either as rule ‘followers’ or rule ‘breakers’. ([12], p. 7)

Additionally, some studies reported that mothers perceived healthcare professionals as unsupportive and, at times, even uncaring [16, 34]. Some mothers further experienced professional interactions as intrusive and degrading, noting that the healthcare focus was often limited to the infant or the mother’s body. This led to feelings of objectification, where mothers felt reduced to mere instrumental functionality, which in turn undermined their sense of worth and confidence in breastfeeding [32].

**Table 2:** Extracted data

Theme	Summary of the theme	Text examples
**Theme 1**. The Ideal of the ‘Good Mother’ Under Pressure	The mothers felt pressure to breastfeed from both society and themselves. They adhered to cultural norms surrounding breastfeeding and valued breast milk above all other feeding methods. Breastfeeding was seen as central to the maternal role, implying that to breastfeed was to be “a good mother”. Ideals strongly influenced the concept of “the good mother”; therefore, learning to breastfeed became crucial to developing mothering skills. Mothers expected breastfeeding to be instinctive, natural, and easy, yet many felt unprepared for its physical and emotional realities. When breastfeeding proved unsuccessful, they described a clash between idealised expectations and lived experience. Many feared judgment for not matching the expected motherhood image and felt redundant as mothers when replacing breast milk with formula.	‘Participants identified societal pressures that were associated with the cultural norms around breastfeeding.’ [16]. ‘Women found that the pressure came from external sources, such as health professionals and other mothers, but also from themselves.’ [16]. ‘It’s more about our fundamental idea of what makes a good woman, what makes a good mother.’ [16]. ‘There is a false belief that breastfeeding is instinctive for mothers. Although it is a natural practice, it has to be learnt and requires training.’ [6]. ‘All the women found their breastfeeding experience difficult and challenging. There was a real sense that they weren’t prepared for the reality of breastfeeding.’ [27]. ‘The women also spoke of the pressure to breastfeed that is put on women by society and, also, by themselves.’ [27]. ‘A consistent finding was that the women described a clash or incongruity between their highly idealized standards and the reality of their early breastfeeding experience.’ [29]. ‘The final theme highlighted the part that breastfeeding played in the maternal role (…) inferring that to breastfeed was to be a good mother.’ [16]. ‘All of the participants in the study described some sort of clash between their expectations and the reality of the experience.’ [29]. ‘For the women in this study the context against which they described their breastfeeding experience was the idealized view of self as “good mother” or “perfect mother”.’ [29]. ‘This concept is shaped by Western society’s view of motherhood, and influenced by women’s expectations for high levels of achievement in all realms of their lives.’ [29]. ‘Pressure and judgement operated as the social, personal and cultural backdrop to many women’s infant feeding decisions and experiences. Women sensed pressure (from professionals, media and social networks) to breastfeed and moral judgement around their feeding decisions.’ [12]. ‘Though most people probably won’t admit it, there are judgements about feeding your infant using formula milk instead of breastfeeding because…it is not the right thing to do’ [24]. ‘I was concerned that other people might think … that I had taken the easy way out. I know it is a crazy thought, but it is a strong feeling of not being a “real” mother’ [24]. ‘Participants also believed that breastfeeding would be effortless because it was widely described as a natural maternal process: “Before my delivery, I expected to breastfeed because I knew that breastfeeding is a natural process”.’ [1]. ‘Before childbirth, mothers described strong expectations that breastfeeding would be natural, instinctive, and easily manageable.’ [1].
**Theme 2**. Existential Consequences Arising from Breastfeeding Difficulties	The life transition of becoming a mother gave rise to profound existential concerns, including feelings of insecurity and vulnerability. Mothers perceived breastfeeding as closely tied to their sense of self-worth and maternal identity. Thus, when they were unable to breastfeed or experienced difficulties doing so, they felt emotionally burdened and at a loss, describing feelings of guilt, shame, self-doubt, sadness, disappointment, and grief, along with a persistent sense of failure; of being an unsuccessful mother to their infant.	‘(…) In spite of all I had suffered, I felt bad. It’s as if you’re a bad mother because you stopped.’ [6]. ‘So you feel vulnerable, you don’t know how to ask or what to do, or what would be best, and you don’t even know if you are doing things right or wrong.’ [6]. ‘For mothers who could not, or struggled to, breastfeed, this could cause emotional problems if they had anticipated being able to breastfeed.’ [16]. ‘I felt it was all tied up in my worth as a mother… this is about whether or not you’re a good mum.’ [16]. ‘The major life change may also give rise to existential issues and sometimes even feelings of depression.’ [32]. ‘The gaze from others reinforces the mother’s own sense of being a failure, a feeling of being an unsuccessful mother to her infant.’ [32]. ‘The majority of the women spoke poignantly about a sense of failure, guilt, disappointment, or shame and self-doubt about not continuing with breastfeeding’ [29]. ‘Nearly all the women reported lingering self-doubts that continued for extended periods of time before any sense of resolution occurred.’ [29]. ‘Some women were able to work through their guilt relatively quickly, consoled by the fact that their infants were thriving and that they had done the best they could. For other mothers, the process of resolving feeling of guilt and recrimination took months or years.’ [29]. ‘I wanted to feel that closeness, and I wanted to feel mothering. And I felt guilty for so long.’ [29]. ‘Managing the situation was associated with cognitive and emotional responses of the women. All of them described more than one response. These included self-doubts and feelings of failure when breastfeeding did not work.’ [36]. ‘It takes a lot out of you when you think ‘Oh, I’m the biggest loser because I just can’t breastfeed.’ [36]. ‘(…) Mothers shared their feelings of regret, sadness or guilt if they wished they had been able to breastfeed their baby longer.’ [23]. ‘I think there was a whole grieving process for me around that, around letting go of that dream.’ [2]. ‘Women struggled with the dissonance between their expectations of breastfeeding and the reality of cessation.’ [2]. ‘The following days, weeks and even months, I felt grief-stricken that the breastfeeding did not work out as I had hoped. And I can still feel this grief, I can actually feel the grief right now as we speak, as I am sitting here with tears in my eyes simply talking about the matter. Also, I felt sad that my body could not do it properly … and, of course, I blamed myself.’ [24].
**Theme 3**. Insufficient Support Undermining Breastfeeding	Mothers perceived the support from healthcare professionals as insufficient and poorly suited to their individual needs. At times, healthcare staff appeared unsupportive or even uncaring, which left mothers feeling emotionally dismissed. Additionally, care was sometimes experienced as intrusive and degrading, with the focus solely directed towards the infant or the body. This narrow focus contributed to feelings of objectification, reducing mothers to a state of instrumental functionality and leaving them feeling worthless and undermined in their ability to breastfeed.	‘The interviewees considered that the support they had received from some health professionals was insufficient or unsuited to their needs.’ [6]. ‘Participants highlighted that at least some health professionals involved in their care were at times unsupportive and came across as uncaring.’ [16]. ‘The care can be experienced as non-caring, for example, intrusive hands-on breastfeeding help, or care that focuses solely on the infant or the body. Such care is degrading in that it objectifies the woman and reduces her to solely an instrumental functionality.’ [32]. ‘(…) Some health professionals employed ‘rules’ in explaining how breastfeeding ought to be performed and how this provided the sense of there being only one correct way to breastfeed is described (…) There was no notion of what might be ‘right’ for one mother might not be ‘right’ for another or that ‘the answer’ might need to be personalised or adapted.’ [12]. ‘Staff had different attitudes and approaches, and some women reported being made to feel guilty or inadequate if their baby did not breastfeed well, or if they chose to give formula. They would have liked help with formula feeding and felt neglected compared with breastfeeding mothers.’ [34]. ‘Midwives and other staff could be perceived as (…) not always kind or supportive.’ [34]. ‘Many mothers complained that they had received conflicting advice, especially during hospitalization. This made insecure mothers feel even more at a loss because they were not in a condition to choose between different kinds of advice.’ [19]. ‘Many women in the study described health professionals as ‘annoying because they kept (…) telling me something different all the time.’ [2]. ‘To me, it seemed as if the healthcare system did not have either the time or the resources to prioritise breastfeeding support… Therefore, the system’s attitude in regard to breastfeeding was like “Well if you can’t breastfeed, then why don’t you just give your infant a bottle instead?” Still, they constantly emphasised the importance and benefits of breastfeeding. However, I never experienced a health professional who really took the time to sit with me and say “Why don’t you try and latch the infant and then I will help you do this properly.’ [24]. ‘However, several mothers described limited or inconsistent professional support when they encountered breastfeeding difficulties. Some participants reported that healthcare providers were often busy or unavailable to pro vide practical breastfeeding assistance: “When I asked the midwife to help me with breastfeeding, she told me to try by myself because the ward was busy”.’ [1]. ‘Others expressed disappointment that they did not receive sufficient guidance regarding breastfeeding techniques such as posi tioning and latching: “The nurses told me breastfeeding is important, but no one showed me how to position the baby properly”.’ [1].

## Discussion

This scoping review mapped the existing knowledge on mothers’ experiences of unwanted early cessation of breastfeeding and suggests that these experiences have a profound emotional impact, to the extent that the mother’s sense of motherhood becomes conflicted.

The first overarching theme, *The Ideal of the ‘Good Mother’ Under Pressure*, highlights how mothers experience motherhood as conflicted in the context of early breastfeeding cessation. Specifically, this review found that mothers encountered a clash between socio-cultural norms, internalised expectations, and the lived reality of breastfeeding, wherein their maternal identity felt threatened. This conflict manifested both in how they perceived society’s judgment and how they viewed themselves. Given that contemporary motherhood ideals are closely intertwined with cultural expectations of successful breastfeeding, deviation from this ideal naturally challenges a mother’s moral and social identity. Societal perceptions often equate breastfeeding success with being a “good mother”, thereby reinforcing the moral dimension of infant feeding choices. Mothers feared being judged for failing to meet idealised expectations of motherhood, often associated with successful breastfeeding. Ceasing breastfeeding earlier than intended contributed to feelings of guilt, shame, and inadequacy. A similar concern is echoed in another study, where mothers experienced emotional discomfort when bottle-feeding in public, suggesting that how they fed their baby became a visible marker of maternal adequacy [20]. Because infant feeding is a public act embedded in cultural meanings, the visibility of the bottle symbolically communicates success or failure in fulfilling maternal norms. Concerning self-perception, this review found that mothers viewed breastfeeding as central to motherhood, implying that breastfeeding was equivalent to being a successful mother. Consequently, when breastfeeding did not proceed as expected, their identity as competent ‘good’ mothers was undermined, weakening their understanding of their own maternal identity. Although not all women internalised these ideals to the same extent, the findings collectively reinforce that early breastfeeding cessation is not merely a practical or psychological issue but a deeply identity-forming experience situated within socio-cultural expectations and personal ideals. Some mothers may rationalise or accept early cessation without severe identity conflict; yet, the prevailing pattern across studies indicates that emotional distress is often relational - arising from conflicting internal and external expectations rather than from cessation itself.

In the second overarching theme, *Existential Consequences Arising from Breastfeeding Difficulties*, this review found that breastfeeding challenges leading to early cessation often triggered grief, shame, and guilt. These findings resonate with previous research showing that such emotions are particularly pronounced among formula-feeding mothers and that guilt and shame significantly predict symptoms of postpartum anxiety and depression [15]. Similarly, several studies reported a discrepancy between maternal ideals of breastfeeding and the lived experience of not succeeding, which has been associated with heightened risks of guilt, shame, depression, and anxiety [7, 22, 35, 37].

Together, these findings highlight that unwanted early cessation of breastfeeding should also be understood in relation to maternal well-being and mental health, rather than solely as an infant-feeding outcome. The emotional impact of stopping breastfeeding can therefore be understood as arising from a perceived violation of maternal duty - an existential rupture rather than a behavioural failure, leaving mothers in an existential crisis. In this sense, unwanted cessation may also shape mothers’ early experiences of caring for, relating to, and becoming a mother to their infant. This interpretation aligns with phenomenological perspectives suggesting that embodied practices, such as breastfeeding, contribute to women’s existential sense of being mothers; when disrupted, these practices threaten ontological security. Consistent with the view, this review also found that mothers experienced a persistent sense of failure - both as women and mothers - related to breastfeeding cessation, affecting their present and future maternal identity [31]. Palmér et al. showed that mothers who experience an unsuccessful breastfeeding trajectory are at risk of developing existential breastfeeding trauma that may shape future feeding decisions. Previous breastfeeding experiences, therefore, have the potential to either enable or hinder subsequent breastfeeding, depending on the emotional meaning attached to them [31]. Because breastfeeding self-efficacy, defined as a mother’s confidence in her ability to breastfeed, is constructed through past experiences, negative trajectories can lower confidence and expectations for future attempts. Kronborg et al. [18] found that limited success during first-time breastfeeding significantly reduced both intention and self-efficacy regarding future breastfeeding, thereby making subsequent efforts more difficult. This provides clear empirical evidence that poor first experiences predict future challenges, underscoring the need for adequate, continuous, and emotionally sensitive early support [18]. Palmér, [31] further highlighted that whether the mother is driven by fear, which threatens future breastfeeding, or by longing, which enables it, depends largely on the quality of professional care received. Hence, support must not only be technically competent but also emotionally attuned - a principle consistent with the WHO’s global recommendations for maternity and newborn services [43].

The third overarching theme, *Insufficient Support Undermining Breastfeeding*, concerns mothers’ experiences of inadequate and emotionally insensitive breastfeeding support and demonstrates how this undermined their ability to sustain breastfeeding. Findings from this review indicate that some mothers perceived healthcare professionals as unsupportive or even uncaring in the context of breastfeeding support. While these perceptions may not reflect professionals’ intentions, they nevertheless shaped mothers’ lived experiences. Several mothers described the care they received as instrumental and poorly tailored to their individual needs. Hamnøy et al. [9] report a similar finding, stating that new mothers need to be perceived and treated as whole individuals – both as breastfeeding mothers and as women with their own needs – to establish and continue breastfeeding, and that inadequate breastfeeding counselling may cause breastfeeding difficulties [9].

Because the postpartum period is physically and emotionally demanding, perceived indifference from professionals can intensify feelings of inadequacy and isolation. The review findings further suggest that new mothers were sometimes regarded as not requiring the same level of care as other patients. Yet, as many mothers were exhausted, hormonally affected, and existentially overwhelmed, being recognised as having legitimate care needs was essential to overcoming breastfeeding difficulties. Allowing mothers to receive care that acknowledges their vulnerability affirms their personhood and fosters trust, both of which are central to recovery and adaptation in the early postpartum phase. Although some mothers undoubtedly received attentive and compassionate care, the pattern across studies indicates that inconsistent or impersonal support often contributed to emotional distress and, in some cases, to the decision to cease breastfeeding. The cumulative evidence, therefore, indicates that postnatal care, including breastfeeding support, would benefit from a shift away from an instrumental, task-oriented model towards a more holistic, relationship-based approach. Given that breastfeeding is an emotionally and culturally embedded experience, support should balance practical guidance with personal sensitivity. This aligns with the WHO’s Protecting, Promoting and Supporting Breastfeeding in Facilities Providing Maternity and Newborn Services guideline, which emphasises the dual importance of technical competence and emotional care [43]. While no single model can meet every mother’s needs, heightened awareness of this complexity should inform both clinical practice and policy development.

This scoping review offers insight into how unwanted early cessation of breastfeeding influences mothers’ experiences of motherhood, often with lasting effects on maternal identity and future breastfeeding trajectories. The review consolidates evidence that early cessation is not an isolated event but an existential, relational, and contextually embedded experience shaped by internal ideals, external expectations, and the quality of professional care. Recognising this complexity can guide professionals towards interventions that acknowledge both the emotional and practical dimensions of breastfeeding. Clinically, this suggests that support should not end when breastfeeding ends, as mothers may continue to need professional support after unwanted early cessation to protect maternal well-being. However, the purpose of this review is not to provide specific strategies for preventing early cessation or ensuring adequate support; rather, its contribution lies in mapping and interpreting mothers’ lived experiences. Therefore, further research, particularly longitudinal and partnership-based studies, is warranted to explore how tailored, empathetic postnatal support can promote maternal well-being and sustained breastfeeding.

### Limitations

This scoping review has some methodological limitations inherent to its design. Although a broad and systematic search strategy was employed, some relevant studies may not have been identified. The inclusion of only English- and Scandinavian-language publications may also have introduced a minor language bias.

In accordance with current scoping review guidance, no formal quality appraisal of the included studies was conducted. This was consistent with the methodological aim of mapping the available literature rather than assessing the quality of evidence. Nevertheless, the absence of a standardised quality assessment should be considered. All included studies were original empirical studies and appeared relevant and methodologically appropriate for addressing the review question. Accordingly, the findings should be understood as a mapping of reported experiences across the included studies, rather than as conclusions based on formally appraised evidence.

### Strengths

This scoping review also has several strengths. To our knowledge, it is the first review to comprehensively explore mothers’ experiences of unwanted early cessation of breastfeeding. Applying the JBI framework for scoping reviews ensured a transparent, systematic, and methodologically rigorous approach. Furthermore, the review synthesised all available research, capturing both the breadth and depth of current knowledge on mothers’ experiences of early breastfeeding cessation.

## Conclusion

This scoping review has mapped existing knowledge on mothers’ experiences of unwanted early cessation of breastfeeding. The findings reveal that this experience is not merely a matter of feeding practice but one that often profoundly shapes mothers’ perceptions of themselves as mothers and influences their broader sense of identity and worth. Mothers’ narratives illuminate how breastfeeding is both culturally and emotionally constructed as a core element of motherhood, one that, when unfulfilled, gives rise to existential consequences such as shame, guilt, grief, and a pervasive sense of failure.

Notably, this review demonstrates that early cessation is experienced within a web of conflicting expectations: between societal ideals of the ‘good mother’, internalised aspirations, and the unpredictable, often challenging realities of breastfeeding. These tensions were further intensified by a perceived lack of tailored, emotionally attuned professional breastfeeding support, which in some cases contributed to mothers’ feelings of inadequacy and emotional distress.

Taken together, the findings underscore the need to reframe breastfeeding support within clinical practice, from a predominantly instrumental model to one that acknowledges the existential and emotional dimensions of early motherhood. Mothers’ experiences highlight the importance of responsive care that validates their emotional needs, affirms their maternal identity, and supports rather than prescribes. Recognising the emotional burden of unwanted early cessation has implications not only for improving maternal wellbeing in the postpartum period but also for enhancing breastfeeding self-efficacy in future pregnancies. As such, healthcare systems must acknowledge the lasting impact of these early experiences and respond with compassion, sensitivity, and structural continuity of care.

## Relevance to clinical practice

This scoping review is the first to provide a comprehensive overview of existing literature on mothers’ experiences of unwanted early breastfeeding cessation. By mapping these experiences, the review deepens our understanding of the emotional processes mothers undergo when ending breastfeeding earlier than intended. The findings provide a comprehensive account of the distress associated with these experiences and offer a sound basis for incorporating such insights into both clinical practice and policy development.

## Supplementary Information

Below is the link to the electronic supplementary material.

Supplementary Material 1

Supplementary Material 2

## Data Availability

The data supporting this scoping review is available upon request from the first author.

## References

[1] Al-Marzouqi M, Al-Dhoani. Experience of primiparous mothers on exclusive breastfeeding in Oman. J Hum Lactation. 2026;1–7. 10.1177/08903344261445161. 10.1177/08903344261445161 42153485

[2] Ayton JE, Tesch L, Hansen E. Women’s experiences of ceasing to breastfeed: Australian qualitative study. BMJ Open. 2019;9(5):e026234. 10.1136/bmjopen-2018-026234. 31064807 10.1136/bmjopen-2018-026234 PMC6528004

[3] Bailey C, Pain RH, Aarvold JE. A ‘give it a go’ breast-feeding culture and early cessation among low-income mothers. Midwifery. 2004;20(3):240–50. 10.1016/j.midw.2003.12.003. 15337280 10.1016/j.midw.2003.12.003

[4] Balogun OO, O’Sullivan EJ, McFadden A, Ota E, Gavine A, Garner CD, Renfrew MJ, MacGillivray S. Interventions for promoting the initiation of breastfeeding. Cochrane Database Syst Rev. 2016;11(11):CD001688. 10.1002/14651858.CD001688.pub3. 27827515 10.1002/14651858.CD001688.pub3 PMC6464788

[5] Birch EE, Garfield S, Castaneda Y, Hughbanks-Wheaton D, Uauy R, Hoffman D. Visual acuity and cognitive outcomes at 4 years of age in a double-blind, randomized trial of long-chain polyunsaturated fatty acid-supplemented infant formula. Early Hum Dev. 2007;83(5):279–84. 10.1016/j.earlhumdev.2006.11.003. 17240089 10.1016/j.earlhumdev.2006.11.003

[6] Cortés-Rúa L, Díaz-Grávalos GJ. Early interruption of breastfeeding. A qualitative study. Enfermería Clínica (English Edition). 2019;29(4):207–15. 10.1016/j.enfcle.2018.11.001. 10.1016/j.enfcli.2018.11.003 30638896

[7] Fallon V, Komninou S, Bennett KM, Halford JCG, Harrold JA. The emotional and practical experiences of formula-feeding mothers. Matern Child Nutr. 2017;13(4). 10.1111/mcn.12392. 10.1111/mcn.12392 PMC6866173 27862970

[8] Feenstra MM, Kirkeby J, Thygesen M, Danbjorg M, D. B., Kronborg H. Early breastfeeding problems: A mixed method study of mothers’ experiences. Sex Reprod Healthc. 2018;16:167–74. 10.1016/j.srhc.2018.04.003. 29804762 10.1016/j.srhc.2018.04.003

[9] Hamnøy IL, Kjelsvik M, Baerug AB, Dahl BM. Breastfeeding mother’s experiences with breastfeeding counselling: a qualitative study. Int Breastfeed J. 2024;19(1):34. 10.1186/s13006-024-00636-x. 38745330 10.1186/s13006-024-00636-x PMC11095000

[10] Hauck YL, Fenwick J, Dhaliwal SS, Butt J. A Western Australian survey of breastfeeding initiation, prevalence and early cessation patterns. Matern Child Health J. 2011;15(2):260–8. 10.1007/s10995-009-0554-2. 20077131 10.1007/s10995-009-0554-2

[11] Horta BL, Victora CG. Short-term effects of breastfeeding: a systematic review on the benefits of breastfeeding on diarrhoea and pneumonia mortality. World Health Organization; 2013. p. 1–54. Available from: https://iris.who.int/bitstream/handle/10665/95585/9789241506120_eng.pdf?sequence=1&isAllowed=y.

[12] Hunt L, Thomson G. Pressure and judgement within a dichotomous landscape of infant feeding: a grounded theory study to explore why breastfeeding women do not access peer support provision. Matern Child Nutr. 2017;13(2). 10.1111/mcn.12279. 10.1111/mcn.12279 PMC6865888 27037727

[13] Jackson KT, Mantler T, OʼKeefe-McCarthy S. Women’s Experiences of Breastfeeding-Related Pain. MCN Am J Matern Child Nurs. 2019;44(2):66–72. 10.1097/nmc.0000000000000508. 30688667 10.1097/NMC.0000000000000508

[14] Jackson L, De Pascalis L, Harrold J, Fallon V. Guilt, shame, and postpartum infant feeding outcomes: A systematic review. Matern Child Nutr. 2021;17(3):e13141. 10.1111/mcn.13141. 33491303 10.1111/mcn.13141 PMC8189225

[15] Jackson L, Fallon V, Harrold JA, De Pascalis L. Psychosocial predictors of post-natal anxiety and depression: Using Structural Equation Modelling to investigate the relationship between pressure to breastfeed, health care professional support, post-natal guilt and shame, and post-natal anxiety and depression within an infant feeding context. Matern Child Nutr. 2024;20(1):e13558. 10.1111/mcn.13558. 37752680 10.1111/mcn.13558 PMC10750005

[16] Keevash J, Norman A, Mortimer S, Ferrario H. What influences women to continue or stop breastfeeding: a thematic analysis. Br J Midwifery. 2018;26. 10.12968/bjom.2018.26.10.651.

[17] Kramer MS, Kakuma R. Optimal duration of exclusive breastfeeding. Cochrane Database Syst Rev. 2012;2012(8):CD003517. 10.1002/14651858.CD003517.pub2. 22895934 10.1002/14651858.CD003517.pub2 PMC7154583

[18] Kronborg H, Foverskov E, Vaeth M, Maimburg RD. The role of intention and self-efficacy on the association between breastfeeding of first and second child, a Danish cohort study. BMC Pregnancy Childbirth. 2018;18(1):454. 10.1186/s12884-018-2086-5. 30466403 10.1186/s12884-018-2086-5 PMC6251224

[19] Kronborg H, Harder I, Hall EO. First time mothers’ experiences of breastfeeding their newborn. Sex Reprod Healthc. 2015;6(2):82–7. 10.1016/j.srhc.2014.08.004. 25998875 10.1016/j.srhc.2014.08.004

[20] Larsen JS, Kronborg H. When breastfeeding is unsuccessful–mothers’ experiences after giving up breastfeeding. Scand J Caring Sci. 2013;27(4):848–56. 10.1111/j.1471-6712.2012.01091.x. 23057626 10.1111/j.1471-6712.2012.01091.x

[21] Laursen MF, Sakanaka M, von Burg N, Morbe U, Andersen D, Moll JM, Pekmez CT, Rivollier A, Michaelsen KF, Molgaard C, Lind MV, Dragsted LO, Katayama T, Frandsen HL, Vinggaard AM, Bahl MI, Brix S, Agace W, Licht TR, Roager HM. Bifidobacterium species associated with breastfeeding produce aromatic lactic acids in the infant gut. Nat Microbiol. 2021;6(11):1367–82. 10.1038/s41564-021-00970-4. 34675385 10.1038/s41564-021-00970-4 PMC8556157

[22] Lee G. Self-discrepancy in mothers: protective factors against maternal guilt, shame, anxiety, and depression [doctoral dissertation]. University of Liverpool; 2021. https://liverpool.idm.oclc.org/login?url?url=https://www.proquest.com/dissertations-theses/self-discrepancy-mothers/protective-factors/docview/2461952450/se-2.

[23] Leurer MD, Misskey E. The Psychosocial and Emotional Experience of Breastfeeding: Reflections of Mothers. Glob Qual Nurs Res. 2015;2:2333393615611654. 10.1177/2333393615611654. 28462320 10.1177/2333393615611654 PMC5342287

[24] Mahon J, Dreyer P. Primiparous women’s experiences of unwanted early cessation of breastfeeding: A qualitative study. Nordic J Nurs Res. 2024;44:20571585241276464. 10.1177/20571585241276464.

[25] Mahon J, Dreyer P, Francis SR. Women’s experiences of unwanted early cessation of breastfeeding: A scoping review protocol. Cent Open Sci. 2024. 10.17605/OSF.IO/ZFMUE. 10.1186/s13006-026-00883-0 PMC13499327 42629586

[26] Mangrio E, Persson K, Bramhagen AC. Sociodemographic, physical, mental and social factors in the cessation of breastfeeding before 6 months: a systematic review. Scand J Caring Sci. 2018;32(2):451–65. 10.1111/scs.12489. 28569436 10.1111/scs.12489

[27] Massov L. Clinically overweight and obese mothers and low rates of breastfeeding: Exploring women’s perspectives. New Z Coll Midwives J. 2015;51:23–9. 10.12784/nzcomjnl51.2015.4.23-29.

[28] Morrison AH, Gentry R, Anderson J. Mothers’ Reasons for Early Breastfeeding Cessation. MCN Am J Matern Child Nurs. 2019;44(6):325–30. 10.1097/NMC.0000000000000566. 31633522 10.1097/NMC.0000000000000566

[29] Mozingo JN, Davis MW, Droppleman PG, Merideth A. It wasn’t working. Women’s experiences with short-term breastfeeding. MCN Am J Matern Child Nurs. 2000;25(3):120–6. 10.1097/00005721-200005000-00004. 10810844 10.1097/00005721-200005000-00004

[30] Neupane S, de Oliveira CVR, Palombo CNT, Buccini G. Association between breastfeeding cessation among under six-month-old infants and postpartum depressive symptoms in Nevada. PLoS ONE. 2024;19(1):e0297218. 10.1371/journal.pone.0297218. 38277396 10.1371/journal.pone.0297218 PMC10817202

[31] Palmér L. Previous breastfeeding difficulties: an existential breastfeeding trauma with two intertwined pathways for future breastfeeding-fear and longing. Int J Qual Stud Health Well-being. 2019;14(1):1588034. 10.1080/17482631.2019.1588034. 30893016 10.1080/17482631.2019.1588034 PMC6442107

[32] Palmér L, Carlsson G, Brunt D, Nystrom M. Existential security is a necessary condition for continued breastfeeding despite severe initial difficulties: a lifeworld hermeneutical study. Int Breastfeed J. 2015;10:17. 10.1186/s13006-015-0042-9. 25960763 10.1186/s13006-015-0042-9 PMC4425864

[33] Peters MDJ, McInerney GC, Baldini P, Soares C. Chapter 11: Scoping reviews. In: Aromataris E, Munn Z, editors. *JBI manual for evidence synthesis*. 2020. https://synthesismanual.jbi. 10.46658/JBIMES-20-12

[34] Redshaw M, Henderson J. Learning the hard way: expectations and experiences of infant feeding support. Birth. 2012;39(1):21–9. 10.1111/j.1523-536X.2011.00509.x. 22369602 10.1111/j.1523-536X.2011.00509.x

[35] Sonnenburg C, Miller YD. Postnatal Depression: The Role of Good Mother Ideals and Maternal Shame in a Community Sample of Mothers in Australia. Sex Roles. 2021;85(11):661–76. 10.1007/s11199-021-01239-0.

[36] Spannhake M, Jansen C, Görig T, Diehl K. It is a very emotional topic for me—managing breastfeeding problems among German mothers: a qualitative approach. Healthcare (Basel). 2021;9(10). 10.3390/healthcare9101352. 10.3390/healthcare9101352 PMC8544576 34683032

[37] Taylor EN, Wallace LE. For Shame: Feminism, Breastfeeding Advocacy, and Maternal Guilt. Hypatia. 2012;27(1):76–98. 10.1111/j.1527-2001.2011.01238.x.

[38] Tomori C, Hernandez-Cordero S, Busath N, Menon P, Perez-Escamilla R. What works to protect, promote and support breastfeeding on a large scale: A review of reviews. Matern Child Nutr 18 Suppl. 2022;3(Suppl 3):e13344. 10.1111/mcn.13344. 10.1111/mcn.13344 PMC9113479 35315573

[39] Tricco AC, Lillie E, Zarin W, O’Brien KK, Colquhoun H, Levac D, Moher D, Peters MDJ, Horsley T, Weeks L, Hempel S, Akl EA, Chang C, McGowan J, Stewart L, Hartling L, Aldcroft A, Wilson MG, Garritty C, Straus SE. PRISMA Extension for Scoping Reviews (PRISMA-ScR): Checklist and Explanation. Ann Intern Med. 2018;169(7):467–73. 10.7326/M18-0850. 30178033 10.7326/M18-0850

[40] UNICEF, World Health Organization. Food & nutrition action in health systems, nutrition and food safety. Global breastfeeding scorecard, 2017: tracking progress for breastfeeding policies and programmes. 2017.https://cdn.who.int/media/docs/default-source/breastfeeding/global-breastfeeding-collective/global-bf-scorecard-2017.pdf?sfvrsn=d5ebb905_5&download=true.

[41] Victora CG, Bahl R, Barros AJ, Franca GV, Horton S, Krasevec J, Murch S, Sankar MJ, Walker N, Rollins NC, Series LB, G. Breastfeeding in the 21st century: epidemiology, mechanisms, and lifelong effect. Lancet. 2016;387(10017):475–90. 10.1016/S0140-6736(15)01024-7. 26869575 10.1016/S0140-6736(15)01024-7

[42] Watkins S, Meltzer-Brody S, Zolnoun D, Stuebe A. Early breastfeeding experiences and postpartum depression. Obstet Gynecol. 2011;118(2 Pt 1):214–21. 10.1097/AOG.0b013e3182260a2d. 21734617 10.1097/AOG.0b013e3182260a2d

[43] World Health Organization. Guideline: protecting, promoting and supporting breastfeeding in facilities providing maternity and newborn services. World Health Organization; 2017. https://www.who.int/publications/i/item/9789241550086. 29565522

[44] World Health Organization. Infant and young child feeding – fact sheets. 2023 [cited 2023 Jun 19]. From https://www.who.int/news-room/fact-sheets/detail/infant-and-young-child-feeding.

[45] World Health Organization, United Nations Children’s Fund, Global Breastfeeding Collective. Rates of breastfeeding increase around the world through improved protection and support. Global breastfeeding scorecard. 2023 [cited 2023 Jun 19]. Available from:https://www.unicef.org/media/150586/file#:~:text=and%202030%20targets-,BREASTFEEDING%20PRACTICES,50%25%20(Figure%201).

[46] Yström E. Breastfeeding cessation and symptoms of anxiety and depression: a longitudinal cohort study. BMC Pregnancy Childbirth. 2012;12:36. 10.1186/1471-2393-12-36. 22621668 10.1186/1471-2393-12-36 PMC3449190

