# Supplementary material for: Mothers’ experiences of unwanted early cessation of breastfeeding: a scoping review
Source: Int Breastfeed J. 2026 Aug 12;21:77. doi: 10.1186/s13006-026-00883-0 (PMC13499327; doi:10.1186/s13006-026-00883-0)
Supplement: Supplementary file 1 [file 13006_2026_883_MOESM1_ESM.docx]

Supplemental material

**Appendix I: Search Strategy**
MEDLINE (PubMed). Search conducted on 19 June 2024, again on 4 July 2025 and again on 25 June 2026.

| Search | Query | Records retrieved |
| --- | --- | --- |
| Search Strategy June 19, 2024 | | |
| #1 | (("Weaning"[MeSH Terms] OR ("Cessation"[Title/Abstract] OR "stop*" [Title/Abstract] OR "quit*"[Title/Abstract] OR "ceas*"[Title/Abstract] OR "wean*"[Title/Abstract] OR "continu*"[Title/Abstract])) AND ("Early"[Title/Abstract] OR "unwanted"[Title/Abstract] OR "involuntarily"[Title/Abstract] OR "involuntary"[Title/Abstract]) AND ("experience*"[Title/Abstract] OR "perception*"[Title/Abstract] OR "attitude*"[Title/Abstract] OR "view*"[Title/Abstract] OR "feel*" [Title/Abstract] OR "perspective*"[Title/Abstract]) AND ("Breastfeeding"[Title/Abstract] OR "Breast Feeding"[Title/Abstract] OR "Breast Feeding"[MeSH Terms])) Filters: Danish, English, Norwegian, Swedish | 671 |
| Search Strategy for 4th July 2025 | | |
| #1 | ((((((((Weaning[MeSH Terms]) OR (Cessation[Title/Abstract])) OR (stop*[Title/Abstract])) OR (quit*[Title/Abstract])) OR (ceas*[Title/Abstract])) OR (continu*[Title/Abstract])) AND ((((Early[Title/Abstract]) OR (unwanted[Title/Abstract])) OR (involuntary[Title/Abstract])) OR (involuntary[Title/Abstract]))) AND ((((((experience*[Title/Abstract]) OR (perception*[Title/Abstract])) OR (attitude*[Title/Abstract])) OR (view*[Title/Abstract])) OR (feel*[Title/Abstract])) OR (perspective*[Title/Abstract]))) AND (((Breastfeeding[Title/Abstract]) OR (Breast Feeding[Title/Abstract])) OR (Breast Feeding[MeSH Terms])) Filters: in the last 1 year, Danish, English, Norwegian, Swedish | 43 |
|  | **Search Strategy for 25 June 2026** |  |
|  | ((((((((Weaning[MeSH Terms]) OR (Cessation[Title/Abstract])) OR (stop*[Title/Abstract])) OR (quit*[Title/Abstract])) OR (ceas*[Title/Abstract])) OR (continu*[Title/Abstract])) AND ((((Early[Title/Abstract]) OR (unwanted[Title/Abstract])) OR (involuntary[Title/Abstract])) OR (involuntary[Title/Abstract]))) AND ((((((experience*[Title/Abstract]) OR (perception*[Title/Abstract])) OR (attitude*[Title/Abstract])) OR (view*[Title/Abstract])) OR (feel*[Title/Abstract])) OR (perspective*[Title/Abstract]))) AND (((Breastfeeding[Title/Abstract]) OR (Breast Feeding[Title/Abstract])) OR (Breast Feeding[MeSH Terms])) Filters: in the last 1 year, Danish, English, Norwegian, Swedish | 72 |
