# Supplementary material for: Mothers’ experiences of unwanted early cessation of breastfeeding: a scoping review
Source: Int Breastfeed J. 2026 Aug 12;21:77. doi: 10.1186/s13006-026-00883-0 (PMC13499327; doi:10.1186/s13006-026-00883-0)
Supplement: Supplementary file 2 [file 13006_2026_883_MOESM2_ESM.docx]

Supplemental material

**Appendix II: PCC**

| Population |  | Concept |  | Context |
| --- | --- | --- | --- | --- |
| Mothers  that have ceased breastfeeding early *(within the first six months postpartum)* | **AND** | Experiences of unwanted early cessation of breastfeeding within the first six months postpartum | **AND** | Breastfeeding |
| Weaning [Free text/MeSH]  OR |  | Attitude* [Free text/MeSH]  **OR** |  | Breastfeeding [Free text/MeSH]  **OR** |
| Cessation [Free text/MeSH]  OR |  | View* [Free text/MeSH]  **OR** |  | “Breast Feeding [Free text/MeSH] |
| Stop*[Free text/MeSH]  OR |  | Feel* [Free text/MeSH]  **OR** |  |  |
| Quit* [Free text/MeSH]  OR |  | Perspective* [Free text/MeSH] **OR** |  |  |
| Ceas* [Free text/MeSH]  OR |  | Experience* **OR** |  |  |
| Wean* [Free text/MeSH]  OR |  | Perception* |  |  |
| Continu* [Free text/MeSH] |  |  |  |  |
| Early [Free text/MeSH]  OR |  |  |  |  |
| Unwanted [Free text/MeSH]  OR |  |  |  |  |
| Involuntarily [Free text/MeSH]  OR |  |  |  |  |
| Involuntary [Free text/MeSH] |  |  |  |  |
